# Optimization of a Biometric System Based on Acoustic Images

**DOI:** 10.1155/2014/780835

**Published:** 2014-01-28

**Authors:** Alberto Izquierdo Fuente, Lara Del Val Puente, Juan J. Villacorta Calvo, Mariano Raboso Mateos

**Affiliations:** ^1^Departamento de Teoría de la Señal y Comunicaciones e Ingeniería Telemática, Universidad de Valladolid, Paseo Belén 15, 47011 Valladolid, Spain; ^2^Departamento de Ciencia de los Materiales e Ingeniería Metalúrgica, Expresión Gráfica de la Ingeniería, Ingeniería Cartográfica, Geodesia y Fotogrametría Ingeniería Mecánica e Ingeniería de los Procesos de Fabricación, Universidad de Valladolid, Paseo del Cauce 59, 47011 Valladolid, Spain; ^3^Facultad de Informática, Universidad Pontificia de Salamanca, Compañia 5, 37002 Salamanca, Spain

## Abstract

On the basis of an acoustic biometric system that captures 16 acoustic images of a person for 4 frequencies and 4 positions, a study was carried out to improve the performance of the system. On a first stage, an analysis to determine which images provide more information to the system was carried out showing that a set of 12 images allows the system to obtain results that are equivalent to using all of the 16 images. Finally, optimization techniques were used to obtain the set of weights associated with each acoustic image that maximizes the performance of the biometric system. These results improve significantly the performance of the preliminary system, while reducing the time of acquisition and computational burden, since the number of acoustic images was reduced.

## 1. Introduction

Biometric identification [1–3] is a subject of active research, where new algorithms and sensors are being developed. The most widely used identification systems are based on fingerprints, hand geometry, retina, face, voice, vein, signature, and so forth. The fusion of information from multiple biometric systems is also improving the performance of identification and verification systems [4].

Radar-based systems require expensive hardware and can be unreliable due to the very low reflection intensity from humans. Acoustic imaging provides a simple and cheap sensor alternative that allows obtaining very precise range and angular information. Particularly, in the acoustic field, there are two accurate and reliable classification systems for targets:

- animal echolocation, performed by mammals such as bats, whales, and dolphins, where nature has developed specific waveforms for each type of task [5, 6] such as the classification of different types of flowers [7];
- acoustic signatures used in passive sonar systems [8, 9], which analyse the signal received by a target in the time-frequency domain.

There are few papers working on acoustic imaging in air for the detection of human beings. Moebus et al. [10, 11] worked with the ultrasonic band (50 kHz) using a 2D array and beamforming in reception. They analysed solid objects (poles and a cuboid on a pedestal) in their first work and human images more recently. They showed that humans have a distinct acoustic signature and proposed to model the echoes from the reflection parts of objects in the scene by a Gaussian mixture-model. Based on the parameters of this model, a detector could be designed to discriminate between person and nonperson objects.

In previous works, the authors of this paper developed multisensor surveillance and tracking systems based on acoustic arrays and image sensors [12, 13]. In November, 2011, authors were working on the development of a novel biometric system, based on acoustic images acquired with electronic scanning arrays [14, 15]. Humans were acoustically scanned by an active system working from 6 to 12 kHz (audioband) that registered their acoustic images. Thus, the system could identify people by comparing the acquired acoustic images with a previously acquired database of said images. This system used beamforming with a linear microphone array and a linear tweeter array in transmission and reception, respectively [16]. This paper was the first one related to acoustic imaging in air for biometric identification of humans in the literature.

That previous work was based on 4 positions and 4 frequencies, and it evaluated the mean square error (MSE) between the acoustic images, assuming that all these images had the same weight in the error calculation and that all the images provided relevant information.

This new work has examined the contribution that each of the images associated with a position and a frequency has in the performance of the biometric system and has optimized the weights associated with the selected images.

On a first phase, the contribution of the acoustic images was analysed, assuming that their weights on the MSE were unitary or null, and working with a variable set of images from only 1 image to up to 16 images. After that, on a second phase, a weight optimization was done on the set of selected acoustic images, so that each image contributed to the calculation of the MSE proportionally to the information provided to the biometric identification between individuals.

In this paper, Section 2 describes the system including its functional description, its hardware architecture, the acoustic array, and the acoustic profiles.  Section 3 describes the results previously published, which, implicitly, use unitary weights for all the images. This section also analyses the contribution of each image either individually or grouped with other images and the system performance by optimizing the weights for the selected images. Finally, Section 4 presents our conclusions.

## 2. Material and Methods

### 2.1. Functional Description

**Figure 1:**
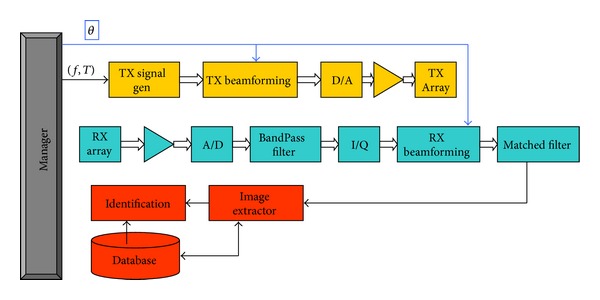
Functional description block diagram.

Based on basic radar/sonar principles [17, 18], an acoustic sound detection and ranging system for biometric identification was proposed [16], according to the block diagram in Figure 1.

This system performed three main tasks: (i) person scanning and detection, (ii) acoustic images acquisition, and (iii) person identification based on a database of acoustic images.

For each steering angle, the system performed: (i) transmission beamforming, (ii) reception beamforming, and (iii) match filtering. After processing all the steering angles, a two-dimensional matrix was formed and stored that this represented the acoustic image.

The application software developed had four operation modes:

- *Channel calibration*. A calibration procedure to ensure that all channels had the same phase and gain [19].
- *Surveillance*. The system detected and estimated the position of the targets in the chamber, visualizing an acoustic image.
- *Image acquisition.* The system captured the acoustic image of a person for a predefined set of frequencies and positions.
- *Biometric identification.* For the person under analysis, the system got the acoustic images and compared them with a set of acoustic images of X individuals, previously stored in a database.

### 2.2. Hardware Architecture

**Figure 2:**
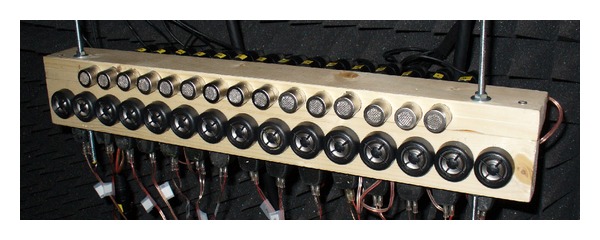
Transmitter and receiver arrays.

The biometric system had four elements:

- a computer with a real-time acquisition system for 16 channels, based on 1.5 M gate FPGA Xilinx Spartan-3 DSP and two Omnibus I/O Daughter Card sites;
- a preamplifier and amplifier system;
- a transmitter (Tx) uniform linear array (ULA) with 15 tweeters and a receiver (Rx) ULA with 15 microphones, as it is shown in Figure 2;
- an acoustic anechoic chamber with a 5 × 3 × 2.5 m working area which was designed for a 500 Hz cutoff frequency.

**Figure 3:**
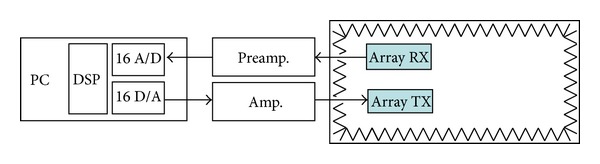
Hardware architecture block diagram.

Figure 3 shows a block diagram of the system and the interconnection between its elements.

### 2.3. Acoustic Array

#### 2.3.1. Spatial Aperture Selection

Two ULAs with 15 *λ*/2-equispaced sensors were employed. These arrays had different spatial apertures in order to reduce sidelobe levels on the final beampattern (Tx + Rx).

A transmission array with a 50 cm spatial aperture and a reception array with a 40 cm spatial aperture were used. On the transmission array, the tweeters were placed so as to occupy the minimum space.

#### 2.3.2. Frequency Band Selection

On the basis of the angular resolution (3-dB beamwidth of the mainlobe), the absence of grating lobes, the frequency response of the microphone-tweeter pair, and the frequency response of a person, four different frequencies that guarantee the independence of the obtained images were selected [16]: 6 kHz (*f*_1_), 8 kHz (*f*_2_), 10 kHz (*f*_3_), and 12 kHz (*f*_4_), where the frequency gap was the maximum in order to obtain independent images. 

The maximum steering angle was determined by the size of the person, his/her distance from the array, and the nonappearance of grating lobes. Based on these considerations, the following parameters were selected:

- the positioning area was located 3 m from the array;
- the maximum width of a person with outstretched arms was 2 m.

**Figure 4:**
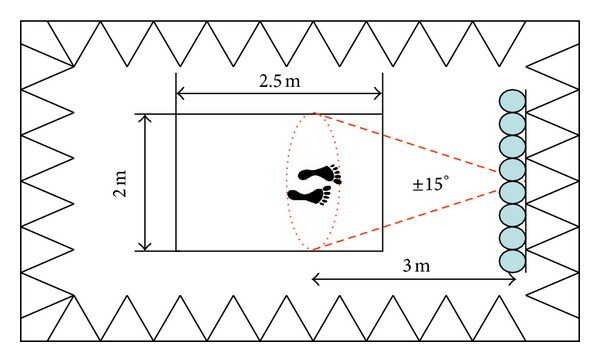
Scanning and positioning area.

Therefore, for the scanning and positioning area, the selected angle excursion was ±15°, as shown in Figure 4.

#### 2.3.3. Angle Resolution Cells and Number of Beams

Given a ULA, Δ*u* is defined as the 3-dB beamwidth of the mainlobe in the sin(*θ*)* space*, where Δ*u* = sinΔ*θ*, having Δ*θ* the 3-dB beamwidth of the mainlobe in degrees. Beamwidth in sin(*θ*)* space* does not depend on the steering angle and, therefore, assuming that beams are 3-dB overlapped, the number of beams necessary to cover the exploration zone will be [20]:

(1) $$\begin{array}{c} M=\text{round }\left(\frac{2\cdot \sin\theta_{\max}}{\Delta u}\right), \end{array}$$

where *θ*_max⁡_ = 15° is the angular excursion.

The number of beams for each frequency, *M*_*k*_, is shown in Table 1.

### 2.4. Acoustic Profiles

Following the previous design considerations, the system retrieved the acoustic image associated with a rectangle of 2 m × 2.5 m (width × depth) dimensions, where the person under analysis had to be located 3m away from the line array, as described in Figure 4.

A 2 ms pulse width and a sampling frequency *f*_*s*_ = 32 kHz were used. This value was a trade-off between range resolution and received energy. The acoustic images were collected from 2.0 m to 4.5 m, in the range coordinate, and from −15° to 15°, in the azimuth coordinate, using *M*_*k*_ steering angles.

**Figure 5:**
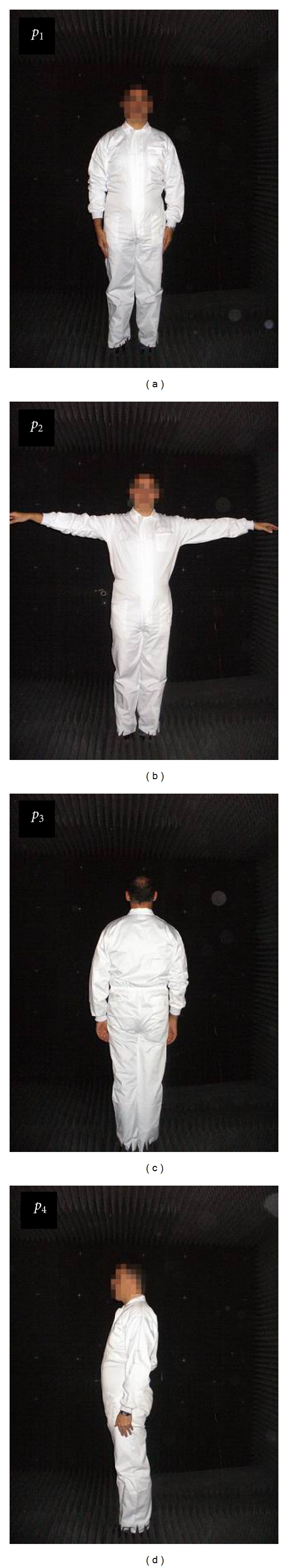
Person positions.

The selected positions for the person under analysis were front view with arms folded on both sides (*p*_1_), front view with arms outstretched (*p*_2_), back view (*p*_3_), and side view (*p*_4_).  Figure 5 shows the four positions using a test subject.

The acoustic profile, *P*_*i*_, associated with person *i*, included the 16 acoustic images obtained for the positions (*p*_1_, *p*_2_, *p*_3_, and *p*_4_), evaluated at the frequencies (*f*_1_, *f*_2_, *f*_3_, and *f*_4_).

**Figure 6:**
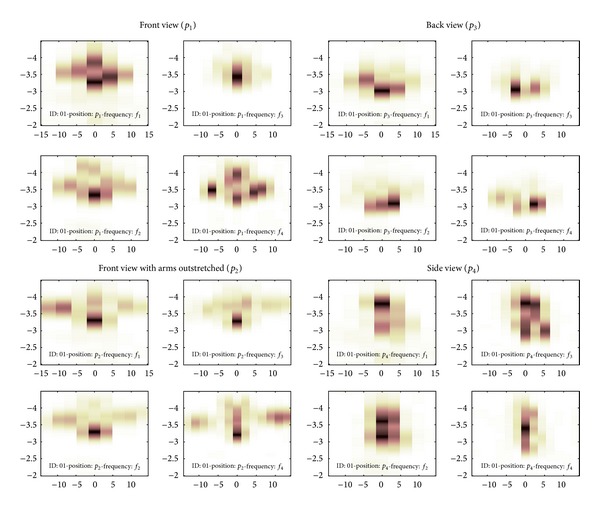
Acoustic images. *x*-axis: angle (degrees); *y*-axis: range (m).

Figure 6 shows the acoustic images for (i) the front view position (*p*_1_) where the head and trunk of the subject can be clearly identified, (ii) the front view position with arms outstretched (*p*_2_) where the head and arms of the subject can be clearly identified, (iii) the back view position (*p*_3_) where the back of the head can be identified, and (iv) the side view position (*p*_4_) where the closest shoulder and side of the head can be identified.

## 3. Results and Discussion

### 3.1. Previous Study: Biometric Identification via Mean Square Error

#### 3.1.1. Metric Based on Mean Square Error (MSE)

The identification implemented by the acoustic biometric system was based on the mean square error (MSE) between acoustic images from two different profiles [21].

First, a function *E*_*p*_^*f*^[*i*, *j*] was defined as the mean square error between an acoustic image *I*_*i*_(*r*, *s*) from profile *P*_*i*_ and an acoustic image *I*_*j*_(*r*, *s*) from profile *P*_*j*_, for a specific frequency *f* and position *p*:

(2) $$\begin{array}{c} E_{p}^{f}\left[i,j\right]=\overset{R}{\underset{r=1}{\sum }}\overset{S}{\underset{s=1}{\sum }}{\left(I_{i}\left(r,s\right)-I_{j}\left(r,s\right)\right)}^{2},\;i,j=1\cdots NP, \end{array}$$

where *I*(*r*, *s*) is a *R* × *S* matrix and *NP* is the number of acoustic profiles stored in the database.

Then, the multifrequency error function *E*_*p*_[*i*, *j*] was defined as the sum of the errors at each frequency for a specific position *p*:

(3) Ep[i,j]=Ep6 kHz[i,j]+Ep8 kHz[i,j]+Ep10 kHz[i,j]+Ep12 kHz[i,j].

Finally, the global error function *E*[*i*, *j*] was defined as the sum of the multifrequency errors at each position *p*:

(4) $$\begin{array}{c} E\left[i,j\right]=E_{p_{1}}^{}\left[i,j\right]+E_{p_{2}}^{}\left[i,j\right]+E_{p_{3}}\left[i,j\right]+E_{p_{4}}\left[i,j\right]. \end{array}$$

If *P*_*k*_ was an unknown profile to be identified, the algorithm associated the profile, *P*_*k*_, to the person “*i*” in the database whose profile *P*_*i*_ had the minimum *E*[*k*, *i*] value. The normalized global error was defined as the distance or metric used by the acoustic biometric system.

#### 3.1.2. False Match Rate (FMR), False Nonmatch Rate (FNMR) and Receiver Operating Characteristic (ROC) Curve

Based on the methodology to characterize a biometric system [22] and assuming that there were no errors in the acquisition, FNMR and FMR parameters were calculated.

False match rate (FMR) is the probability of the system matching incorrectly the input acoustic profile to a nonmatching template in the database. It measures the percent of invalid inputs which are incorrectly accepted. Thus, FMR was obtained by matching acoustic profiles of different people.

The global error *E*[*i*, *j*] was calculated for all these cases. And then the FMR parameter was calculated as the percentage of matching whose error value was equal or less than distance *d*:

(5) $$\begin{array}{c} E\left[i,j\right]\leq d, \end{array}$$

where distance *d* is the set of possible values of the global error.

False nonmatch rate (FNMR) is the probability of the system not matching the input acoustic profile to a matching template in the database. It measures the percent of valid inputs which are incorrectly rejected. Hence, FNMR was obtained by matching acoustic profiles of the same people.

Again, the normalized global error was calculated for all these cases. Then the FNMR parameter was calculated as the percentage of matching whose error value was greater or equal than distance *d*:

(6) $$\begin{array}{c} E\left[i,j\right]\geq d. \end{array}$$

A receiver operating characteristic (ROC) curve, is a graphical plot which illustrates the performance of a classifier system as its discrimination threshold, distance *d* in this case, is varied. This ROC curve is a visual characterization of the trade-off between the FNMR and the FMR obtained. It was created by plotting the FMR values versus the FNMR values, at various threshold/distance settings.

#### 3.1.3. Test Scenario

This acoustic biometric system, based on an electronic scanning array using sound detection and ranging techniques, was analysed in order to find the feasibility of employing acoustic images of a person as a biometric feature.

In this previous study [16], 10 people (5 men and 5 women with different morphological features, as shown in Table 2) were scanned in the four selected positions with a narrow acoustic beam, employing four pulsed tone signals, with the selected frequencies.

To evaluate this system, acoustic profiles were captured 10 times for each of the 10 people under test during 10 days. In the analysis, all people wore an overall, as common reference clothing, in order to eliminate clothing as a distinctive factor.

**Figure 7:**
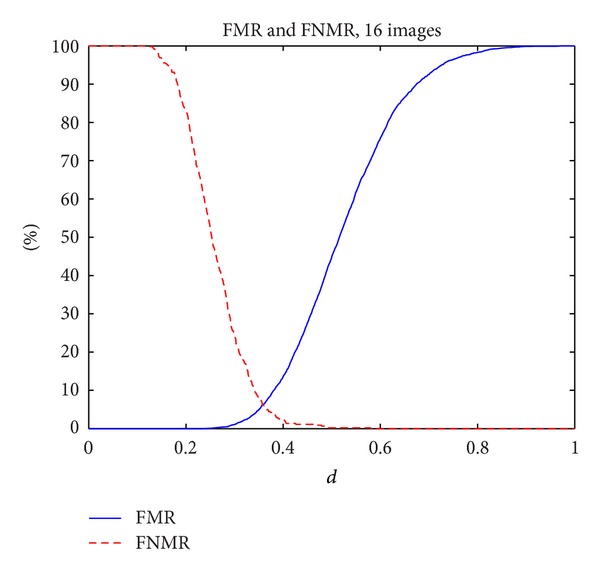
Functions FMR and FNMR versus distance *d*.

Figure 7 shows the FMR and the FNMR functions versus the normalized distance *d* obtained in the analysis.

It can be observed that the value of the equal error rate (EER)—the crossing point between FMR and FNMR functions—was 6.22%, for a distance *d* = 0.35 m.

**Figure 8:**
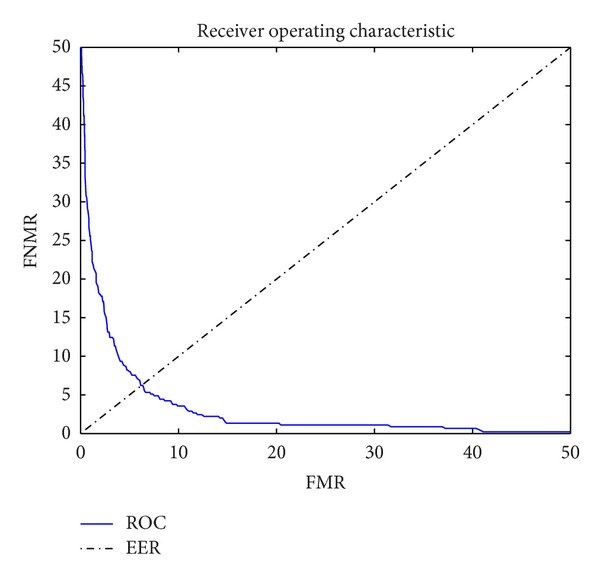
ROC.

The corresponding ROC curve is shown in Figure 8.

The FNMR, FMR, and ROC curves obtained were comparable to those of commercial biometric systems, confirming the feasibility of using acoustic images in biometric systems.

### 3.2. Contribution of the Images to the Acoustic Profile

The global error *E*[*i*, *j*] used in Section 3.1.1 can be reformulated as the sum of the errors due to each acoustic image associated with a frequency and a position:

(7) $$\begin{array}{c} E\left[i,j\right]=\overset{4}{\underset{f=1}{\sum }}\overset{4}{\underset{p=1}{\sum }}E_{p}^{f}\left[i,j\right]. \end{array}$$

Generalizing this expression, the weighted global error *E*_*w*_[*i*, *j*], where the contribution associated with each image is weighted by a value *w*_*p*_^*f*^, can be defined according to the following expression:

(8) $$\begin{array}{c} E_{w}\left[i,j\right]=\overset{4}{\underset{f=1}{\sum }}\overset{4}{\underset{p=1}{\sum }}w_{p}^{f}E_{p}^{f}\left[i,j\right], \end{array}$$

where the weights are defined between 0 and 1:

(9) $$\begin{array}{c} 0\leq w_{p}^{f}\leq 1. \end{array}$$

For the case where all the images contribute to a unitary weight, the global error coincides with the weighted global error:

(10) $$\begin{array}{c} E\left[i,j\right]=E_{w}\left[i,j\right],\;w_{p}^{f}=1\;\;\forall p\;\;\forall f. \end{array}$$

An analysis to determine if all the images contribute equally in determining the ROC curve of the biometric system was performed. The hypothesis was that there will be images (associated with a position and a frequency) that provide more information than others. The goal was twofold: on the one hand, to detect the most relevant frequencies/positions and, on the other hand, to reduce the complexity of the system by eliminating those frequencies/positions that provided less information.

At this point, the information that an image provided had to be evaluated not only individually but also collectively to establish which images provided supplementary information. The ultimate goal was to obtain the set of images that allowed us to minimize the EER value associated with the system, taking the corresponding value using the global error as a reference and where all images contributed to unitary weights.

To evaluate the different hypotheses, a weight *w*_*p*_^*f*^ = 1—to select an image—and a weight *w*_*p*_^*f*^ = 0—not to select it—were defined.

The following studies were carried out:

- system analysis using a single image;
- system analysis using all the images associated with a position;
- system analysis using all the images associated with a frequency;
- system analysis discarding all the images associated with a position;
- system analysis discarding all the images associated with a frequency;
- System analysis discarding any *N* images.

#### 3.2.1. Individual Images

In this case, the individual information—corresponding to a frequency *f*_*i*_ and a position *p*_*i*_—that each acoustic image provided to the biometric system was analysed, assuming that the rest of the images were not present. In order to achieve this objective, a unitary weight was assigned to the image that corresponds to the selected position and frequency, while the rest of the images had null weights.

The result was equivalent to a biometric system consisting only of an acoustic image. Calculating the EER value of the system for each of the images, the following results were obtained, as shown in Table 3.

It can be checked that the obtained values for each case were very different, resulting in a minimum value of EER = 16.61, for *p*_2_ position (front with arms outstretched) evaluated at frequency *f*_2_ (8 kHz) and a maximum value of the EER = 35.66, for *p*_1_ position (front) evaluated at frequency *f*_3_ (10 kHz). The ratio between EER maximum and minimum values was 2.14.

These results also highlighted that the images associated with position *p*_2_ (front with arms outstretched) were the ones that provide the most information and, on the other hand, images associated with position *p*_1_ (front) and *p*_3_ (back) were those which provide the least information, since they were the columns that had higher EER values.

It became clear that each type of images provided different information and, therefore, it was not reasonable to assign all images the same contribution/weight to the error function.

It was also verified that the EER value for a single acoustic image was far superior to the value obtained when the 16 images were combined with unit weights (EER = 6.22). This indicated that a single image was not enough to constitute a biometric system based on acoustic signatures and that the combination of various frequencies/positions was essential to improve the system performance.

However, when minimizing the complexity of the system, the number of positions and frequencies was a relevant parameter. So, it was of great interest to determine whether the information associated with a position or a frequency provided more or less information than the remaining positions/frequencies. Therefore, the following two sections show the analysis of the performance of the system when using all the images associated with a frequency or a position.

#### 3.2.2. Images Associated with a Position

In this case, the joint information of the 4 images associated with a specific position, assuming that the rest of the images were not present, was analysed. This was achieved by assigning a unitary weight to those images corresponding to position *p*_*i*_ and a null weight to the rest of the images.

The result was equivalent to a biometric system consisting only of 4 acoustic images. Calculating the EER value of the system for each of the positions, the following results were obtained, as shown in Table 4.

This gave a minimum value of the EER = 9.52 for position *p*_2_ (front with arms outstretched) and a maximum value of EER = 25.80 for position *p*_1_ (front). The ratio between EER maximum and minimum values was 2.71.

It was evident that the use of 4 images associated with different frequencies improved substantially the EER values of the individual case. However, it was surprising that the EER value using a single image at position *p*_2_ (the one associated with frequency *f*_2_ = 8 kHz) was lower than some values obtained using 4 images (associated with positions *p*_1_ or *p*_3_).

Clearly, there were significant differences in the information associated with the different spatial positions.

#### 3.2.3. Images Associated with a Frequency

In this case, the joint information of the 4 images associated with a specific frequency, assuming that the rest of the images were not present, was discussed. For this case, a unitary weight was assigned to the images corresponding to the frequency *f*_*i*_ and a null weight to the rest of the images.

The result was equivalent to a biometric system formed only by 4 acoustic images. Calculating the EER of the system for each of the frequencies, the following results were obtained, as shown in Table 5.

A minimum value of the EER = 13.78 for frequency *f*_1_ (6 kHz) and a maximum value of the EER = 17.56 for frequency *f*_3_ (10 kHz) were obtained. The ratio between EER maximum and minimum values was 1.27.

These results showed that using 4 images associated with different positions substantially improved the EER values of the individual case. In this case, the value of EER using a single image for the position *p*_2_ (16.61) presented a value that was equivalent to the EER value using 4 images (13.78 –17.56).

Clearly, there were no significant differences in the information associated with the different frequencies. An EER = 9.52 using 4 frequencies for the position *p*_2_ (front with arms outstretched) was obtained, clearly better than the EER = 13.78 using 4 positions for the frequency *f*_1_ (6 kHz).

The EER values obtained with 4 images were superior to the EER values obtained using all the 16 images, so it was necessary to extend the information by increasing the number of images.

In the next two sections, 12 images were used, discarding the images that correspond to a particular position or frequency.

#### 3.2.4. Images Discarding a Position

This case analysed the information from 12 images associated with three of the four positions, assuming that the rest of the images were not present. A null weight was assigned to the images corresponding to the position discarded, *p*_*i*_, and a unitary weight to the rest of the images.

The result was equivalent to a biometric system consisting only of 12 acoustic images. Calculating the EER of the system for each of the cases, the following results were obtained, as shown in Table 6.

A minimum value of the EER = 5.79, excluding position *p*_1_ (front), and a maximum value of the EER = 12.55, excluding position *p*_2_ (front with arms outstretched), were obtained. Clearly, there were significant differences associated with the discarded positions (EER maximum and minimum ratio = 2.16).

The first conclusion was that better results can be obtained with 12 images (EER = 5.79) than with 16 images (EER = 6.22). Therefore, there were images associated with positions that clearly provided information that degraded the biometric system, rather than providing information to improve it.

In view of the previous results, position *p*_1_ (front) was not significant in the presence of the information obtained from positions *p*_2_, *p*_3_, and *p*_4_. It seemed evident that the system could remove the images associated with position *p*_1_ in order to reduce its complexity.

As a second conclusion, in relation to the results obtained using 4 images associated with a position, except for the combination that excludes *p*_2_, working with 12 images improved the performance of the biometric system. Note that the above combination did not use position *p*_2_, which was shown to be the one that contributed to the most information to the system. 

#### 3.2.5. Images Discarding a Frequency

This case analysed the information from 12 images associated with three of the four frequencies, assuming that the rest of the images were not present. A null weight was assigned to the images corresponding to the frequency discarded, *f*_*i*_, and a unitary weight to the rest of the images.

The result was equivalent to a biometric system formed only by 12 acoustic images. Calculating the EER of the system for each of the frequencies, the following results were obtained, as shown in Table 7.

A minimum value of the EER = 7.34, excluding frequency *f*_1_ (6 kHz), and a maximum value of the EER = 8.68, excluding frequency *f*_3_ (10 kHz) were obtained. Clearly the differences associated with the discarded frequencies were of little significance (EER maximum and minimum ratio = 1.18).

In this case, with 12 images (EER = 7.34), the system did not work better than with 16 images (EER = 6.22). Therefore, the use of multiple frequencies upgraded the biometric features of the system.

#### 3.2.6. Discarding *N* Images

In view of the results, it was interesting to analyse the behaviour of the system when *N* images were discarded, where *N* was any number between 1 and 14. In preliminary studies, 15 images, 12 images, and 4 images were discarded but always grouped by frequency or by position.

In principle, discarding images means a reduction of information, which should be reflected as an increase in EER. However, in the previous section, it was shown that discarding 4 images associated with a position provided the best results. If this process of elimination of any frequency and position was generalized, lower EER values could be obtained.

**Figure 9:**
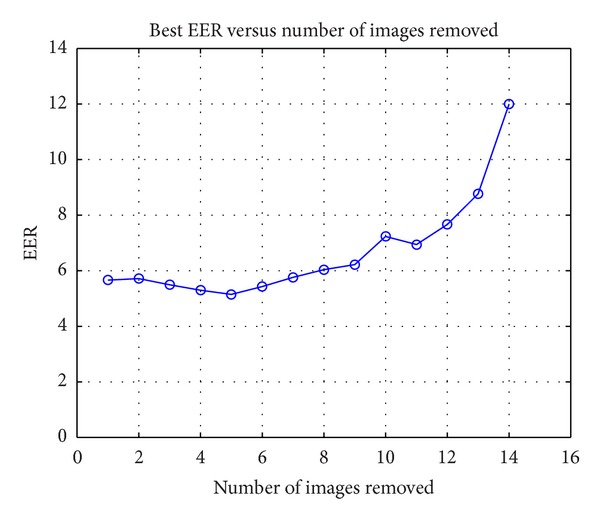
Equal error rate versus number of removed images.

This study was carried out to obtain the results shown in Figure 9.

The EER value had a minimum for the case *N* = 5, where the two combinations with lower EER were selected. For these two cases the images included/discarded are presented in Tables 8 and 9.

Given these results, and since the difference in the value of EER was small, the case with a value of EER = 5.19 was the selected candidate. This case allowed the complete elimination of all the images of position *p*_1_ and, therefore, simplified the capturing of images of the person from 4 positions to 3. This represented a 25% reduction in acquisition time and in storage space.

By analysing the case *N* = 4 the EER function had a minimum value of 5.29. The two combinations with the smallest EER values were selected. Their results are shown in Tables 10 and 11.

Note that if combination number 4, which eliminated position *p*_1_, was selected, a value of EER = 5.79, higher than the selected for *N* = 5, could be obtained. On the other hand, removing *p*_3_-*f*_4_ image improved the quality of the system, since both for *N* = 4 and for *N* = 5 the candidates with lower EER values did not include this image.

In conclusion, combination number 2 was selected with a value of EER = 5.19.

### 3.3. Weight Optimization

If, instead of quantifying the weights with unitary or null values, the value of the weights was optimized to minimize the weighted global error *E*_*w*_[*i*, *j*], a value of EER lower than the results of the previous section could be obtained.

The goal was to obtain the weights that minimized the weighted global error, defined by

(11) $$\begin{array}{c} {\min(E_{w}\left[i,j\right])|}_{w_{p}^{f}}=\overset{4}{\underset{f=1}{\sum }}\overset{4}{\underset{p=1}{\sum }}w_{p}^{f}E_{p}^{f}\left[i,j\right]. \end{array}$$

Solving this optimization problem was complex because it was a multivariate optimization problem whose computational burden grew exponentially with the number of variables or weights.

The analysis of the total number of possible combinations required a very high computational cost in the order of *C* ^16^, where *C* was the number of different discretized weight values, making the problem directly unfeasible. Considering the results of Section 4, the number of weights to be optimized could be reduced from 16 to 11, decreasing the computational burden, although the process time was still too high.

A preliminary analysis of the error function indicated that it was a nonconcave space with multiple local minima, so those algorithms based on the technique of the gradient could not be used. In practice, an optimization algorithm based on Powell's method [23] was used. This algorithm was based on directional searches and recursion and it significantly reduced the computational burden.

Firstly, the 11 weights associated with the images that were selected in the previous section were optimized. After that, the optimization with 16 images was performed in order to verify whether the exclusion of images had reduced the system performance.

#### 3.3.1. Optimization with 11 Images

The obtained results yielded a value of EER = 4.17. The optimal vector of weights is shown in Table 12.

Then, in order to validate whether the deleted information contained in the 5 discarded images could improve the biometric performance of the system, an optimization was carried out for the 16 images.

#### 3.3.2. Optimization with 16 Images

The obtained results yielded a value of EER = 4.00. The optimal vector of weights is shown in Table 13.

Note that the weights associated with position *p*_1_ were much lower than the weights for the other positions. This validates the hypothesis that the data associated with this position provided very little information to the biometric system.

Since the EER value obtained with 16 images was lower than the EER value achieved with 11 images, the next step was to analyse whether increasing the number of images could improve the performance of the system.

#### 3.3.3. Optimization with 12 Images

In this case, multiple combinations were tested, obtaining a value of EER = 4.0 for the case that discarded all the images of position *p*_1_, as shown in Table 14.

It is not necessary to analyse the results of a larger number of images, since in this case with *N* = 12 images, the obtained EER value was equivalent to the case of *N* = 16. Therefore, discarded images did not provide meaningful information to the biometric system.

Optimal vectors for *N* = 12 and *N* = 16 were quite different. However, in both cases, the net information was the same, due to the fact that the value of the obtained EER was equivalent. Using 16 images, the information was redundant and therefore the information could be distributed among multiple images. But, using 12 images the information could only be obtained from the 12 selected images.

In any case, it should be noted that there were multiple combinations of weights which lead to the same value of EER for a fixed number of images. This fact showed that the function has multiple minima, as it was a very complex error surface.

**Figure 10:**
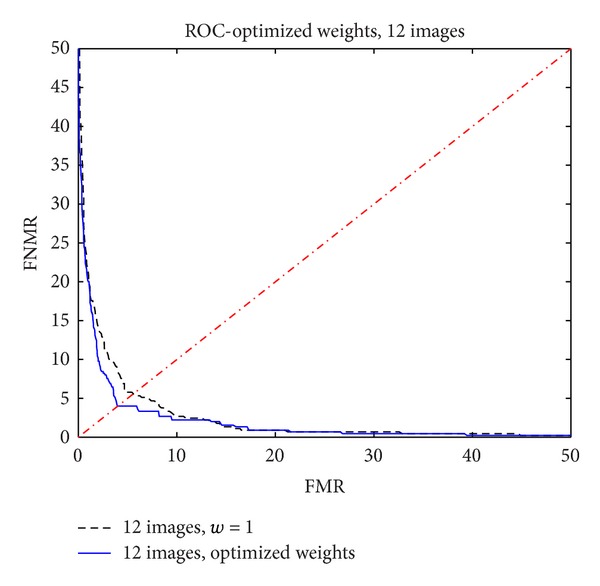
ROC function with 12 images: unitary weights versus optimized weights.

Figure 10 shows the ROC functions for the case of *N* = 12 with optimized weights compared to the case *N* = 12 with unitary weights.

It is observed that the optimization process had significantly improved the performance of the biometric system.

**Figure 11:**
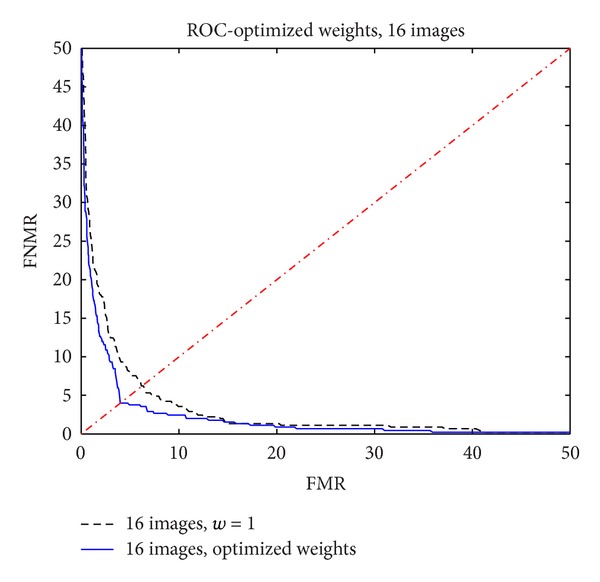
ROC function with 16 images: unitary weights versus optimized weights.

In a similar way, Figure 11 shows the ROC functions for the case of *N* = 16 with optimized weights compared to the case *N* = 16 with unitary weights, previously published and summarized in Section 3.1.

Again, it can be observed that the optimization process had improved the performance of the biometric system.

**Figure 12:**
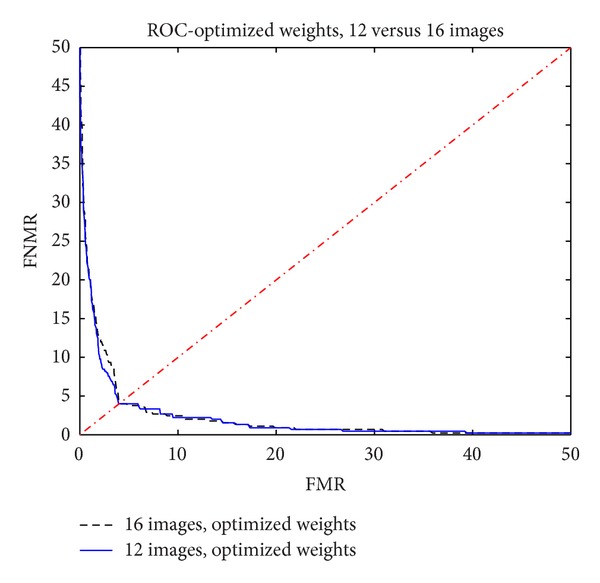
ROC function with 12 images versus 16 images with optimized weights.

Finally, Figure 12 shows ROC functions for the case of *N* = 12 with optimized weights, comparing it to the case *N* = 16, also with optimized weights.

It is observed that the performance obtained with 12 images was equivalent to the one obtained with 16 images.

This study highlighted that the selection of 12 images along with optimization techniques allowed a substantial improvement in the performance of the biometric system while reducing the number of images required.

The original biometric system using 16 images and unitary weights yielded a value of EER = 6.22, and the new system using 12 images and optimized weights yielded a value of EER = 4.00. There was an improvement of over 30%.

## 4. Conclusions

Based on the results obtained in a preliminary publication, where 16 acoustic images of a person—working with 4 frequencies and 4 positions—were used, a methodology for the selection of the most significant images in the face of the biometric system performance was developed.

Each acoustic image that is associated with a position and a frequency provides and shares information that allows to discriminate people from each other.

On a first stage, the contribution of each acoustic image to the biometric system was analysed, assuming that all the images had a unitary or a null weight. We reached the conclusion that with 11 images we can obtain the same performance that with the 16 images. In addition, the images associated with the front position (*p*_1_) are those that provide less information, since much of it can be obtained from the images of the remaining positions. This analysis was carried out measuring the value of EER and selecting an increasing number of images, until the value of EER was minimized.

Afterwards, on a second stage, weights for 11 images were optimized, where the EER value obtained was close to the one obtained optimizing 16 images. We arrived at the conclusion that using 12 acoustic images, which correspond to the positions front with arms outstretched, side and back, the minimum value of the EER can be obtained. This EER value coincides with the value obtained for 16 images.

On the basis of the developed methodology, the selection of acoustic images made on the first stage reduced the number of images and, therefore, significantly reduced the computational burden of the optimization. It was confirmed that the selected acoustic images are essentially the images that must be included in the optimization stage.

Currently, the research group is analyzing the system performance using new frequencies and new metrics not based on MSE.

## Figures and Tables

**Table 1:** Number of beams versus frequency.

*F* (Hz)	Δθ (degrees)	Δ*u*	*M*_*k*_
6000	4.20°	0.0732	7
8000	3.20°	0.0558	9
10000	2.56°	0.0447	11
12000	2.12°	0.0370	13

**Table 2:** Morphological features.

Properties			
ID	Gender	Constitution	Height
00	Male	Very strong	Tall
01	Male	Strong	Average
02	Male	Strong	Average
03	Male	Thin	Tall
04	Male	Normal	Tall
05	Female	Thin	Tall
06	Female	Strong	Small
07	Female	Thin	Average
08	Female	Strong	Average
09	Female	Normal	Small

**Table 3:** Equal error rate using 1 image.

EER-1 image				
Frequency	*p*_1_ (front)	*p*_2_ (front + arms)	*p*_3_ (back)	*p*_4_ (side)
*f*_1_ (6 kHz)	30.38	16.97	31.81	23.93
*f*_2_ (8 kHz)	33.44	**16.61**	28.67	27.09
*f*_3_ (10 kHz)	**35.66**	20.96	33.33	26.28
*f*_4_ (12 kHz)	34.60	16.00	33.89	26.44

**Table 4:** Equal error rate using 4 images associated with a position.

EER-4 images				
	*p*_1_ (front)	*p*_2_ (front + arms)	*p*_3_ (back)	*p*_4_ (side)
*f*_1_ + *f*_2_ + *f*_3_ + *f*_4_	**25.80**	**9.52**	24.25	16.52

**Table 5:** Equal error rate using 4 images associated with a frequency.

EER-4 images				
	*f*_1_ (6 kHz)	*f*_2_ (8 kHz)	*f*_3_ (10 kHz)	*f*_4_ (12 kHz)
*p*_1_ + *p*_2_ + *p*_3_ + *p*_4_	**13.78**	15.44	**17.56**	14.16

**Table 6:** Equal error rate using 12 images, discarding a position.

EER-12 images				
	*p*_2_ + *p*_3_ + *p*_4_	*p*_1_ + *p*_3_ + *p*_4_	*p*_1_ + *p*_2_ + *p*_4_	*p*_1_ + *p*_2_ + *p*_3_
*f*_1_ + *f*_2_ + *f*_3_ + *f*_4_	**5.79**	**12.55**	7.94	8.19

**Table 7:** Equal error rate using 12 images, discarding a frequency.

EER-12 images				
	*f*_2_ + *f*_3_ + *f*_4_	*f*_1_ + *f*_3_ + *f*_4_	*f*_1_ + *f*_2_ + *f*_4_	*f*_1_ + *f*_2_ + *f*_3_
*p*_1_ + *p*_2_ + *p*_3_ + *p*_4_	8.07	**7.34**	**8.68**	7.70

**Table 8:** Equal error rate with 5 images removed (combination number 1).

EER = 5.14															
*p*_1_				*p*_2_				*p*_3_				*p*_4_			
*f*_1_	*f*_2_	*f*_3_	*f*_4_	*f*_1_	*f*_2_	*f*_3_	*f*_4_	*f*_1_	*f*_2_	*f*_3_	*f*_4_	*f*_1_	*f*_2_	*f*_3_	*f*_4_
1	0	0	0	0	1	1	1	1	1	1	0	1	1	1	1

**Table 9:** Equal error rate with 5 images removed (combination number 2).

EER = 5.19															
*p*_1_				*p*_2_				*p*_3_				*p*_4_			
*f*_1_	*f*_2_	*f*_3_	*f*_4_	*f*_1_	*f*_2_	*f*_3_	*f*_4_	*f*_1_	*f*_2_	*f*_3_	*f*_4_	*f*_1_	*f*_2_	*f*_3_	*f*_4_
0	0	0	0	1	1	1	1	1	1	1	0	1	1	1	1

**Table 10:** Equal error rate with 4 images removed (combination number 3).

EER = 5.29															
*p*_1_				*p*_2_				*p*_3_				*p*_4_			
*f*_1_	*f*_2_	*f*_3_	*f*_4_	*f*_1_	*f*_2_	*f*_3_	*f*_4_	*f*_1_	*f*_2_	*f*_3_	*f*_4_	*f*_1_	*f*_2_	*f*_3_	*f*_4_
0	1	0	0	1	1	1	1	1	1	1	0	1	1	1	1

**Table 11:** Equal error rate with 4 images removed (combination number 4).

EER = 5.79															
*p*_1_				*p*_2_				*p*_3_				*p*_4_			
*f*_1_	*f*_2_	*f*_3_	*f*_4_	*f*_1_	*f*_2_	*f*_3_	*f*_4_	*f*_1_	*f*_2_	*f*_3_	*f*_4_	*f*_1_	*f*_2_	*f*_3_	*f*_4_
0	0	0	0	1	1	1	1	1	1	1	1	1	1	1	1

**Table 12:** Equal error rate with 11 images with optimized weights.

EER = 4.17															
*p*_1_				*p*_2_				*p*_3_				*p*_4_			
*f*_1_	*f*_2_	*f*_3_	*f*_4_	*f*_1_	*f*_2_	*f*_3_	*f*_4_	*f*_1_	*f*_2_	*f*_3_	*f*_4_	*f*_1_	*f*_2_	*f*_3_	*f*_4_
**0**	**0**	**0**	**0**	0.35	0.80	0.64	0.35	0.51	0.64	0.13	**0**	0.99	0.71	0.30	0.51

**Table 13:** Equal error rate with 16 images with optimized weights.

EER = 4.00															
*p*_1_				*p*_2_				*p*_3_				*p*_4_			
*f*_1_	*f*_2_	*f*_3_	*f*_4_	*f*_1_	*f*_2_	*f*_3_	*f*_4_	*f*_1_	*f*_2_	*f*_3_	*f*_4_	*f*_1_	*f*_2_	*f*_3_	*f*_4_
0.05	0.02	0.06	0.07	0.63	0.70	0.26	0.49	0.46	0.52	0.17	0.38	0.65	0.52	0.13	0.39

**Table 14:** Equal error rate with 12 images with optimized weights.

EER = 4.00															
*p*_1_				*p*_2_				*p*_3_				*p*_4_			
*f*_1_	*f*_2_	*f*_3_	*f*_4_	*f*_1_	*f*_2_	*f*_3_	*f*_4_	*f*_1_	*f*_2_	*f*_3_	*f*_4_	*f*_1_	*f*_2_	*f*_3_	*f*_4_
**0**	**0**	**0**	**0**	0.51	0.59	0.36	0.26	0.30	0.44	0.11	0.15	0.92	0.52	0.20	0.31
